# Association of acyl-CoA oxidase-like gene polymorphisms with risk, onset age, and beta-cell function of type 1 diabetes in Chinese

**DOI:** 10.3389/fendo.2025.1551159

**Published:** 2025-09-25

**Authors:** Jinhan Liu, Ying Xia, Zhiguo Xie, Xia Li, Gan Huang, Jingyi Hu, Zhiguang Zhou

**Affiliations:** National Clinical Research Center for Metabolic Diseases, Key Laboratory of Diabetes Immunology (Central South University), Ministry of Education, and Department of Metabolism and Endocrinology, The Second Xiangya Hospital of Central South University, Changsha, Hunan, China

**Keywords:** Chinese, type 1 diabetes, genetics, ACOXL, SNP

## Abstract

**Introduction:**

Genome-wide association studies in Caucasians suggested an association between the acyl-CoA oxidase-like (*ACOXL*) gene and type 1 diabetes (T1D). We investigated if polymorphisms in *ACOXL* conferred susceptibility to T1D in Chinese and how they affected the clinical characteristics of T1D.

**Methods:**

MassARRAY was performed in this case–control study to genotype rs4849165 and rs4849135 of *ACOXL* in a collection of 1,280 patients with T1D and 1,331 non-diabetic subjects.

**Results:**

The minor allele C of rs4849165 was associated with an increased risk of T1D (*P* = 0.0013, OR 1.21). Moreover, individuals with the C/C genotype exhibited significantly lower postprandial C-peptide levels compared with T allele carriers (*P* = 0.0058, OR 1.76). The minor allele T of rs4849135 was associated with a decreased risk for T1D (*P =* 0.0098, OR 0.85) and a lower likelihood of having a low level of postprandial C-peptide (*P =* 0.0213, OR 0.78). Patients with G/T or T/T genotypes were more likely to be diagnosed during adulthood than those with G/G genotype (*P* = 0.0206, OR 0.77). No correlation with glutamic acid decarboxylase antibodies (GADA), protein tyrosine phosphatase antibodies (IA-2A), or zinc transporter 8 antibodies (ZnT8A) could be reported here.

**Discussion:**

Polymorphisms in *ACOXL* were associated with susceptibility to T1D in Chinese and showed associations with age at onset and beta-cell function. These loci, together with other genetic signals, may contribute to the development of risk models to identify genetically susceptible individuals in the Chinese population.

## 1 Introduction

In general, type 1 diabetes (T1D) is considered as a chronic disorder in which beta-cells of pancreatic islet are destroyed by autoimmune response, and susceptibility to the disease can be determined by both environmental and genetic factors (1). Besides the HLA complex explaining nearly half of genetic susceptibility to T1D (1), more than 80 non-HLA variants that add moderate risk for T1D individually have been revealed in prior genome-wide association studies (GWASs) and meta-analyses (2–4). Additionally, these genomic locations have already been applied to clinical use, as the genetic risk model consisting of 67 single-nucleotide polymorphisms (SNPs) including both HLA and non-HLA information has proved its efficacy in discriminating subtypes of adult-onset diabetes and newborn screening (5).

Nevertheless, the associations with T1D mentioned above are primarily derived from Caucasian origin and cannot be simply extrapolated to other ethnic groups—for example, the HLA *DR9* haplotype prevalent in patients from East Asia was seen at a considerably low frequency in Caucasians (6, 7). Furthermore, the C1858T of *PTPN22* well known in Caucasian patients with T1D could rarely be found in Chinese (8). Therefore, the remarkable heterogeneity of T1D etiopathogenesis observed among multiple racial groups emphasizes the need to scrutinize the genetics of this disease in underrepresented populations. In terms of Chinese patients, only a few of the genetic variants have been classified as candidate genes for T1D, such as HLA region, *CTLA4*, *PTPN22*, *STAT4*, and *ERBB3*, while a large part of non-HLA genes susceptible to T1D in Caucasians remain unidentified in our population (7–11).

Members of the acyl-CoA oxidase family, such as *ACOX1* and *ACOX2*, are ubiquitously expressed in human tissues, and their roles in peroxisomal β-oxidation have been well characterized (12). The protein encoded by acyl-CoA oxidase-like (*ACOXL*) gene is preferentially expressed in lung, parathyroid gland, urinary bladder, and male sex organs including testis and prostate (The Human Protein Atlas). The function of this protein remains poorly defined, though it is predicted to enable acyl-CoA oxidase activity, fatty acid binding activity, and the maintenance of lipid homeostasis (gene database of NCBI). Variants within *ACOXL* have been engaged in the etiology of various immune-related or metabolic disorders, including chronic lymphocytic leukemia (13), alopecia areata (14), syndromic obesity, and type 2 diabetes (15, 16). Gene polymorphism rs4849135, an intron of *ACOXL*, was initially identified as related to T1D in Caucasians by a dense genotyping array, namely, the Immunochip (4). As far as previous publications are concerned, the role for this or other polymorphisms of *ACOXL* in the pathogenesis of T1D among non-Caucasian participants have not been verified so far.

The current research was thus conducted to investigate if polymorphisms in *ACOXL* conferred risk for T1D in Chinese and if these polymorphisms modified the clinical characteristics of T1D patients, including the positivity of islet autoantibodies, age at diagnosis as well as beta-cell function. In addition to the previously identified T1D risk locus rs4849135, we also selected rs4849165 for analysis. Rs4849165 is located in an intron of *ACOXL*, is not in linkage disequilibrium with rs4849135 (*r* ^2^ <0.20 in the Asian population of the 1000 Genomes Project), and tags 132 SNPs of *ACOXL*. The inclusion of rs4849165 allowed us to capture broader genetic variation within *ACOXL* and to provide a more comprehensive evaluation of its potential role in the Chinese population.

## 2 Materials and methods

### 2.1 Study participants

A total of 1,280 T1D subjects were identified from the Department of Metabolism and Endocrinology in the Second Xiangya Hospital, and they were supposed to fulfill the following inclusion criteria: (1) diagnosed as diabetes under the criteria by WHO in 1999 and (2) acute disease manifestation and dependency of insulin administration upon diagnosis, (3) positive for at least one of the three autoantibodies against pancreatic islet: glutamic acid decarboxylase antibody (GADA), protein tyrosine phosphatase antibody (IA-2A) as well as zinc transporter 8 antibody (ZnT8A). A total of 1,331 healthy controls with normal glucose tolerance but without family history for diabetes were enrolled from the Department of Physical Examination in the Second Xiangya Hospital. All of the study participants declared themselves as of Chinese Han origin. The current research was approved by the Ethics Committee of the Second Xiangya Hospital and was carried out in accordance with the Declaration of Helsinki guidelines. The participants or guardians of T1D children gave their written informed consent and were fully aware of the study process.

### 2.2 Biochemical assays and genotyping methods

Both fasting and postprandial C-peptide levels were determined with methods of chemiluminescence from Adiva Centaur Systemkit. Nearly 70% of the case subjects took this measurement within 12 months after disease onset, whereas the other patients were examined with a disease duration >12 months. Disease duration was included as a covariate in our analyses to account for the progressive decline of C-peptide after T1D onset. Radioligand assays in duplicate permitted the detection of autoantibodies against pancreatic islet, including GADA, IA-2A as well as ZnT8A. All T1D subjects took the examination of autoantibodies at disease onset, and positive results were further validated by a repetition to ensure that cases negative for all autoantibodies and those with ambiguous positivity were not recruited. MassARRAY (Sequenom, the US) was performed to genotype rs4849165 and rs4849135 in *ACOXL* followed by a quality control which achieved call rates of 99.8% and 99.5% for the two polymorphisms respectively.

### 2.3 Statistical analysis

All of the data were computed in SPSS version 22.0 (IBM, USA). We displayed continuous variables with normal distributions as mean ± standard deviation and compared them via Student’s *t*-test, whereas those exhibiting skewed distributions were expressed as median with interquartile range and compared using Mann–Whitney *U*-test. We showed categorical variables as *n* (%) and compared them using chi-square test, which also permitted the test for Hardy–Weinberg equilibrium. Stepwise logistic regression was performed to compute all of the following analyses. When a dominant model was assumed, homozygotes of minor allele and heterozygotes were encoded as 1 and those homozygous for a major allele as 0. Under the assumption of recessive model, those homozygous for a minor allele were set as 1 and heterozygotes in combination with homozygotes of major allele as 0. In the additive model which calculated the impact for each copy of the minor allele, major allele homozygotes were defined as 0, heterozygotes as 1, and minor allele homozygotes as 2. All dominant, recessive, and additive models were adjusted for age to account for the mismatch between cases and controls. When interrogating the clinical relevance of *ACOXL* polymorphisms, outcome variables such as positivity of islet autoantibodies, early disease onset, and lower C-peptide levels were denoted as 1 and the negative results of islet autoantibodies, late T1D onset, and higher C-peptide concentrations as 0. For the analyses of C-peptide levels, disease duration was adjusted for in the logistic regression models.

## 3 Results

### 3.1 Sample description

The demographics and clinical features of 1,280 T1D subjects and 1,331 healthy controls enrolled in this study are listed in **Table 1**. In brief, these two groups showed comparable proportions of male participants (*P =* 0.9247), while the T1D subjects were younger (*P <* 0.0001). Not surprisingly, patients with T1D appeared leaner (*P <* 0.0001) and exhibited remarkably higher concentrations of both fasting plasma glucose (*P <* 0.0001) and 2-h postprandial plasma glucose (*P <* 0.0001) in contrast to non-diabetic subjects. The median age at T1D diagnosis was 21.0 (12.0–34.0) years, and 1,010 patients (86.5%) presented diabetic ketoacidosis at disease onset. The T1D patients had an average HbA1c of 10.52% ± 3.67%, and their median fasting C-peptide and median postprandial C-peptide were 82.90 (29.60–167.00) pmol/L and 165.12 (55.46–341.04) pmol/L, respectively. Disease duration was recorded at the time of C-peptide measurement. At the occurrence of T1D, 90.2%, 49.4%, and 34.5% of patients tested positive for GADA, IA-2A, and ZnT8A, respectively.

**Table 1:** Clinical features of T1D subjects and control subjects.

Characteristics	Control subjects *n*=1,331	T1D subjects *n*=1,280	*P*
Male	712 (53.5)	672 (52.5)	0.9247
Age	39.9 ± 15.6	25.5 ± 15.1	<0.0001
BMI	22.99 ± 3.41	19.06 ± 3.62	<0.0001
FPG (mmol/L)	4.94 ± 0.61	10.20 ± 5.56	<0.0001
PPG (mmol/L)	5.62 ± 1.18	16.15 ± 7.44	<0.0001
Onset age (years)		21.0 (12.0-34.0)	
Diabetic ketoacidosis		1010 (86.5)	
HbA1c (%)		10.52 ± 3.67	
Disease duration (month)		3.0 (0.5-15.0)	
FCP (pmol/L)		82.90 (29.60-167.00)	
PCP (pmol/L)		165.12 (55.46-341.04)	
GADA positivity		1153 (90.2)	
IA-2A positivity		494 (49.4)	
ZnT8A positivity		298 (34.5)	

Sex and positivity of autoantibodies against islet were displayed as *n* (%). The age at diagnosis, FCP, and PCP were denoted as median with interquartile range, whereas the other variables were denoted as mean ± standard deviation.

T1D, type 1 diabetes; BMI, body mass index; FPG, fasting plasma glucose; PPG, 2-h postprandial plasma glucose; HbA1c, glycosylated hemoglobin; FCP, fasting C-peptide; PCP, 2-h postprandial C-peptide; GADA, glutamic acid decarboxylase autoantibody; IA-2A, protein tyrosine phosphatase autoantibody; ZnT8A, zinc transporter 8 autoantibody.

### 3.2 Frequency distributions of genotypes and alleles

Neither the genotypes of rs4849165 (*P =* 0.7586 for T1D group and *P =* 0.1657 for control group) nor those of rs4849135 (*P =* 0.1560 for T1D group and *P =* 0.7391 for control group) showed a significant deviation from Hardy–Weinberg equilibrium. The genotypic and allelic frequency distributions of the two polymorphisms within *ACOXL* are listed in **Table 2**. As for rs4849165, a significantly different distribution of genotypes was seen between the two groups of participants (*P =* 0.0038) and the minor allele C increased risk for T1D (*P =* 0.0013, odds ratio [OR] 1.21, 95% confidence interval [CI] 1.08–1.36). After adjusting for age, the association with T1D risk was best explained by an additive model, where the minor allele C conferred increased risk (*P*_adj_ = 0.0162, OR_adj_ 1.18, 95% CI 1.03–1.34). The initial associations observed in the dominant (*P*_adj_ = 0.0630) and recessive models (*P*_adj_ = 0.0297) were no longer statistically significant after age adjustment and correction for multiple testing.

**Table 2:** Genotypic and allelic frequency distributions of *ACOXL* gene polymorphisms between patients with T1D and healthy controls in a Chinese population.

ACOXL gene polymorphisms	Control subjects	T1D patients	*P*	OR (95% CI)	*P*_adj_	OR_adj_ (95% CI)
rs4849165	*n*=1,327	*n*=1,280				
Genotypes			0.0038	NA	–	
T/T	654 (49.3)	566 (44.2)				
T/C	570 (43.0)	574 (44.8)				
C/C	103 (7.8)	140 (10.9)				
Alleles			0.0013	1.21 (1.08–1.36)	–	
T	1,878 (70.8)	1,706 (66.6)				
C	776 (29.2)	854 (33.4)				
Dominant model			0.0096	1.27 (1.05–1.43)	0.0630	1.18 (0.99–1.40)
T/T	654 (49.3)	566 (44.2)				
T/C+C/C	673 (50.7)	714 (55.8)				
Recessive model			0.0053	1.46 (1.12–1.91)	0.0297	1.39 (1.03–1.87)
T/T+T/C	1,224 (92.2)	1,140 (89.1)				
C/C	103 (7.8)	140 (10.9)				
Additive model						
T/T encoded as 0	654 (49.3)	566 (44.2)	0.0012	1.22 (1.08–1.37)	0.0162	1.18 (1.03–1.34)
T/C encoded as 1	570 (43.0)	574 (44.8)				
C/C encoded as 2	103 (7.8)	140 (10.9)				
rs4849135	*n*=1,321	*n*=1,278				
Genotypes			0.0277	NA	–	
G/G	665 (50.3)	710 (55.6)				
G/T	541 (41.0)	472 (36.9)				
T/T	115 (8.7)	96 (7.5)				
Alleles			0.0098	0.85 (0.75–0.96)	–	
G	1,871 (70.8)	1,892 (74.0)				
T	771 (29.2)	664 (26.0)				
Dominant model			0.0078	0.81 (0.70–0.95)	0.0241	0.82 (0.69–0.97)
G/G	665 (50.3)	710 (55.6)				
G/T+T/T	656 (49.7)	568 (44.4)				
Recessive model			0.2653	0.85 (0.64–1.13)	0.1315	0.79 (0.57–1.08)
G/G+G/T	1,206 (91.3)	1,182 (92.5)				
T/T	115 (8.7)	96 (7.5)				
Additive model			0.0108	0.86 (0.76–0.96)	0.0163	0.85 (0.74–0.97)
G/G encoded as 0	665 (50.3)	710 (55.6)				
G/T encoded as 1	541 (41.0)	472 (36.9)				
T/T encoded as 2	115 (8.7)	96 (7.5)				

Data are shown as *n* (%). Frequencies of genotypes and alleles were compared using chi-square test. Dominant, recessive, and additive models were analyzed using logistic regression, with all models adjusted for age to account for the mismatch between cases and controls. To correct for multiple testing, *P*-values <0.025 were considered statistically significant.

NA, not available.

In terms of rs4849135, the genotypic distribution of the T1D group marginally differed from that in the control group (*P =* 0.0277), the significance of which could not be sustained after correction for multiple testing. The minor allele T was associated with lower odds of T1D (*P =* 0.0098, OR 0.85, 95% CI 0.75–0.96), and this effect remained significant after adjusting for age in both the additive model (*P*_adj_ =0.0163, OR_adj_ 0.85, 95% CI 0.74–0.97) and the dominant model (*P*_adj_ = 0.0241, OR_adj_ 0.82, 95% CI 0.69–0.97). Given the frequencies and OR of the minor alleles, the significance level of 0.05, and the sample size of 2,607, the power was 0.89 for rs4849165 and 0.77 for rs4849135, respectively.

### 3.3 Correlations between genotypes and phenotypes

We went on to analyze the status of the three islet autoantibodies (**Table 3**), early or late disease onset (**Table 4**) as well as levels of C-peptide (**Table 5**) with information split by genotypes of *ACOXL* gene polymorphisms. Whichever genetic model selected, we reported no significant changes in autoantibody status, including GADA, IA-2A, and ZnT8A that could be attributed to the alteration in genotype frequency of rs4849165 or rs4849135 (shown in **Table 3**, all *P*-values >0.05).

**Table 3:** Islet autoantibodies’ status stratified by genotypes of ACOXL polymorphisms in Chinese patients with T1D.

ACOXL gene polymorphisms	Negativity of islet autoantibodies	Positivity of islet autoantibodies	P	OR (95% CI)
rs4849165				
	GADA (-), *n*=125	GADA (+), *n*=1,153		
Dominant model			0.9456	0.99 (0.68–1.43)
T/T	55 (44.0)	511 (44.3)		
T/C+C/C	70 (56.0)	642 (55.7)		
Recessive model			0.6937	0.89 (0.50–1.58)
T/T+T/C	110 (88.0)	1,028 (89.2)		
C/C	15 (12.0)	125 (10.8)		
Additive model			0.8132	0.97 (0.73–1.28)
T/T encoded as 0	55 (44.0)	511 (44.3)		
T/C encoded as 1	55 (44.0)	517 (44.8)		
C/C encoded as 2	15 (12.0)	125 (10.8)		
	IA-2A (-), *n*=505	IA-2A (+), *n*=494		
Dominant model			0.4793	1.09 (0.85–1.41)
T/T	230 (45.5)	214 (43.3)		
T/C+C/C	275 (54.5)	280 (56.7)		
Recessive model			0.4106	1.18 (0.80–1.75)
T/T+T/C	452 (89.5)	434 (87.9)		
C/C	53 (10.5)	60 (12.1)		
Additive model			0.3602	1.09 (0.91–1.31)
T/T encoded as 0	230 (45.5)	214 (43.3)		
T/C encoded as 1	222 (44.0)	220 (44.5)		
C/C encoded as 2	53 (10.5)	60 (12.1)		
	ZnT8A (-), *n*=567	ZnT8A (+), *n*=298		
Dominant model			0.8598	1.03 (0.77–1.36)
T/T	249 (43.9)	129 (43.3)		
T/C+C/C	318 (56.1)	169 (56.7)		
Recessive model			0.9571	1.01 (0.65–1.57)
T/T+T/C	503 (88.7)	264 (88.6)		
C/C	64 (11.3)	34 (11.4)		
Additive model			0.8754	1.02 (0.82–1.25)
T/T encoded as 0	249 (43.9)	129 (43.3)		
T/C encoded as 1	254 (44.8)	135 (45.3)		
C/C encoded as 2	64 (11.3)	34 (11.4)		
rs4849135				
	GADA (-), *n*=125	GADA (+), *n*=1,151		
Dominant model			0.7970	0.95 (0.66–1.38)
G/G	68 (54.4)	640 (55.6)		
G/T+T/T	57 (45.6)	511 (44.4)		
Recessive model			0.5694	0.83 (0.43–1.59)
G/G+G/T	114 (91.2)	1,066 (92.6)		
T/T	11 (8.8)	85 (7.4)		
Additive model			0.6602	0.94 (0.70–1.25)
G/G encoded as 0	68 (54.4)	640 (55.6)		
G/T encoded as 1	46 (36.8)	426 (37.0)		
T/T encoded as 2	11 (8.8)	85 (7.4)		
	IA-2A (-), *n*=505	IA-2A (+), *n*=493		
Dominant model			0.1316	0.83 (0.64–1.06)
G/G	269 (53.3)	286 (58.0)		
G/T+T/T	236 (46.7)	207 (42.0)		
Recessive model			0.7964	0.94 (0.58–1.51)
G/G+G/T	467 (92.5)	458 (92.9)		
T/T	38 (7.5)	35 (7.1)		
Additive model			0.1947	0.88 (0.72–1.07)
G/G encoded as 0	269 (53.3)	286 (58.0)		
G/T encoded as 1	198 (39.2)	172 (34.9)		
T/T encoded as 2	38 (7.5)	35 (7.1)		
	ZnT8A (-), *n*=567	ZnT8A (+), *n*=297		
Dominant model			0.6685	0.94 (0.71–1.25)
G/G	314 (55.4)	169 (56.9)		
G/T+T/T	253 (44.6)	128 (43.1)		
Recessive model			0.9309	0.98 (0.57–1.69)
G/G+G/T	526 (92.8)	276 (92.9)		
T/T	41 (7.2)	21 (7.1)		
Additive model			0.7079	0.96 (0.77–1.20)
G/G encoded as 0	314 (55.4)	169 (56.9)		
G/T encoded as 1	212 (37.4)	107 (36.0)		
T/T encoded as 2	41 (7.2)	21 (7.1)		

Data are shown as *n* (%) and computed by logistic regression. To correct for multiple testing, *P*-values <0.025 were considered statistically significant.

**Table 4:** Age at diagnosis stratified by genotypes of *ACOXL* polymorphisms in Chinese patients with T1D.

ACOXL gene polymorphisms	Early disease onset	Late disease onset	P	OR (95% CI)
rs4849165				
	Onset age <15, *n*=434	Onset age ≥15, *n*=840		
Dominant model			0.1495	1.19 (0.94–1.50)
T/T	179 (41.2)	382 (45.5)		
T/C+C/C	255 (58.8)	458 (54.5)		
Recessive model			0.6557	0.92 (0.63–1.34)
T/T+T/C	389 (89.6)	746 (88.8)		
C/C	45 (10.4)	94 (11.2)		
Additive model			0.3845	1.08 (0.91–1.29)
T/T encoded as 0	179 (41.2)	382 (45.5)		
T/C encoded as 1	210 (48.4)	364 (43.3)		
C/C encoded as 2	45 (10.4)	94 (11.2)		
	Onset age <18, *n*=543	Onset age ≥18, *n*=731		
Dominant model			0.0662	1.23 (0.99–1.55)
T/T	223 (41.1)	338 (46.2)		
T/C+C/C	320 (58.9)	393 (53.8)		
Recessive model			0.6166	1.10 (0.77–1.56)
T/T+T/C	481 (88.6)	654 (89.5)		
C/C	62 (11.4)	77 (10.5)		
Additive model			0.1073	1.15 (0.97–1.36)
T/T encoded as 0	223 (41.1)	338 (46.2)		
T/C encoded as 1	258 (47.5)	316 (43.2)		
C/C encoded as 2	62 (11.4)	77 (10.5)		
rs4849135				
	Onset age <15, *n*=434	Onset age ≥15, *n*=838		
Dominant model			0.0460	0.79 (0.62–1.00)
G/G	258 (59.4)	449 (53.6)		
G/T+T/T	176 (40.6)	389 (46.4)		
Recessive model			0.3217	0.79 (0.50–1.25)
G/G+G/T	406 (93.5)	771 (92.0)		
T/T	28 (6.5)	67 (8.0)		
Additive model			0.0476	0.83 (0.69–1.00)
G/G encoded as 0	258 (59.4)	449 (53.6)		
G/T encoded as 1	148 (34.1)	322 (38.4)		
T/T encoded as 2	28 (6.5)	67 (8.0)		
	Onset age <18, *n*=541	Onset age ≥18, *n*=731		
Dominant model			0.0206	0.77 (0.61–0.96)
G/G	321 (59.3)	386 (52.8)		
G/T+T/T	220 (40.7)	345 (47.2)		
Recessive model			0.2446	0.77 (0.50–1.19)
G/G+G/T	506 (93.5)	671 (91.8)		
T/T	35 (6.5)	60 (8.2)		
Additive model			0.0212	0.81 (0.68–0.97)
G/G encoded as 0	321 (59.3)	386 (52.8)		
G/T encoded as 1	185 (34.2)	285 (39.0)		
T/T encoded as 2	35 (6.5)	60 (8.2)		

Data are shown as *n* (%) and computed by logistic regression. To correct for multiple testing, *P*-values <0.025 were considered statistically significant.

**Table 5:** C-peptide levels stratified by genotypes of *ACOXL* polymorphisms in Chinese patients with T1D.

ACOXL gene polymorphisms	Lower C-peptide levels	Higher C-peptide levels	P	OR (95% CI)
rs4849165				
	FCP < 50 pmol/L, *n*=419	FCP ≥ 50 pmol/L, *n*=758		
Dominant model			0.8828	0.98 (0.76–1.26)
T/T	186 (44.4)	331 (43.7)		
T/C+C/C	233 (55.6)	427 (56.3)		
Recessive model			0.3328	1.22 (0.82–1.80)
T/T+T/C	368 (87.8)	678 (89.4)		
C/C	51 (12.2)	80 (10.6)		
Additive model			0.7282	1.03 (0.86–1.25)
T/T encoded as 0	186 (44.4)	331 (43.7)		
T/C encoded as 1	182 (43.4)	347 (45.8)		
C/C encoded as 2	51 (12.2)	80 (10.6)		
	PCP < 100 pmol/L, *n*=376	PCP ≥ 100 pmol/L, *n*=688		
Dominant model			0.7679	1.04 (0.80–1.36)
T/T	165 (43.9)	313 (45.5)		
T/C+C/C	211 (56.1)	375 (54.5)		
Recessive model			0.0058	1.76 (1.18–2.63)
T/T+T/C	320 (85.1)	624 (90.7)		
C/C	56 (14.9)	64 (9.3)		
Additive model			0.1255	1.17 (0.96–1.42)
T/T encoded as 0	165 (43.9)	313 (45.5)		
T/C encoded as 1	155 (41.2)	311 (45.2)		
C/C encoded as 2	56 (14.9)	64 (9.3)		
rs4849135				
	FCP < 50 pmol/L, *n*=419	FCP ≥ 50 pmol/L, *n*=756		
Dominant model			0.0887	0.80 (0.62–1.03)
G/G	240 (57.3)	408 (54.0)		
G/T+T/T	179 (42.7)	348 (46.0)		
Recessive model			0.0409	0.58 (0.35–0.98)
G/G+G/T	395 (94.3)	691 (91.4)		
T/T	24 (5.7)	65 (8.6)		
Additive model			0.0280	0.80 (0.65–0.98)
G/G encoded as 0	240 (57.3)	408 (54.0)		
G/T encoded as 1	155 (37.0)	283 (37.4)		
T/T encoded as 2	24 (5.7)	65 (8.6)		
	PCP < 100 pmol/L, *n*=376	PCP ≥ 100 pmol/L, *n*=686		
Dominant model			0.0365	0.75 (0.57–0.98)
G/G	222 (59.0)	367 (53.5)		
G/T+T/T	154 (41.0)	319 (46.5)		
Recessive model			0.1101	0.65 (0.39–1.10)
G/G+G/T	352 (93.6)	626 (91.3)		
T/T	24 (6.4)	60 (8.7)		
Additive model			0.0213	0.78 (0.63–0.96)
G/G encoded as 0	222 (59.0)	367 (53.5)		
G/T encoded as 1	130 (34.6)	259 (37.8)		
T/T encoded as 2	24 (6.4)	60 (8.7)		

Data are shown as *n* (%) and computed by logistic regression. To correct for multiple testing, *P*-values <0.025 were considered statistically significant. Disease duration was adjusted for in all of the analyses displayed in this table. Low fasting C-peptide (FCP < 50 pmol/L) was considered absolute insulin deficiency.

FCP, fasting C-peptide; PCP, 2-h postprandial C-peptide.

As shown in **Table 4**, rs4849165 did not modify the age at T1D diagnosis, regardless of genetic models assumed or the cutoff value of onset age selected (all *P*-values >0.05 for rs4849165). When T1D subjects were classified as either those with childhood onset (1–14 years) or those without (>15 years), the T allele of rs4849135 was marginally related to late disease onset (additive model, *P =* 0.0476, OR 0.83, 95% CI 0.69–1.00), and a slightly higher frequency of late onset was observed in T allele carriers in relation to homozygotes of G (68.8% vs. 63.5% in the dominant model, *P =* 0.0460, OR 0.79, 95% CI 0.62–1.00). Both associations lost their significance after Bonferroni correction. However, when the cutoff point for onset age was set as 18 years, the T allele of rs4849135 was significantly associated with adult onset of T1D (additive model, *P =* 0.0212, OR 0.81, 95% CI 0.68–0.97), and a markedly higher percentage of adult onset was found in patients with G/T or T/T genotypes than those with G/G genotype (61.1% vs. 54.6% in the dominant model, *P =* 0.0206, OR 0.77, 95% CI 0.61–0.96). In the recessive model, similar proportions of late disease onset were seen in homozygotes of minor allele T and those with major allele G (*P =* 0.2446).

When both postprandial C-peptide and fasting C-peptide were measured to evaluate the beta-cell function of T1D patients and the adjustment for disease duration was made (**Table 5**), individuals carrying C/C genotypes of rs4849165 were more likely to have low levels of postprandial C-peptide (PCP) compared with T allele carriers (46.7% vs. 33.9% in recessive model, *P =* 0.0058, OR 1.76, 95% CI 1.18–2.63). The detrimental effect of rs4849165 on beta-cell function was not seen in the association analysis of fasting C-peptide (FCP) (*P >*0.05 for all of the three models). Regarding rs4849135, the T allele was associated with a lower likelihood of low FCP levels (additive model, *P =* 0.0280, OR 0.80, 95% CI 0.65–0.98), and patients with T/T genotype were at a slightly lower risk for absolute insulin deficiency (FCP <50 pmol/L) compared to those carrying G allele (27.0% vs. 36.4% in the recessive model, *P =* 0.0409, OR 0.58, 95% CI 0.38–0.98). The T allele significantly decreased the risk for failure of beta-cell function (PCP < 100 pmol/L) in the additive model (*P =* 0.0213, OR 0.78, 95% CI 0.63–0.96), whereas a slightly smaller proportion of patients with T allele had failure of beta-cell function compared to those homozygous for G allele (dominant model, *P =* 0.0365, OR 0.75, 95% CI 0.57–0.98). Nonetheless, the T/T genotype was not significantly associated with PCP in contrast to G/G plus G/T genotype (recessive model, *P =* 0.1101).

## 4 Discussion

As a supplement for the previously identified T1D-susceptible loci in Chinese like HLA complex, *CTLA4*, *PTPN22*, *STAT4*, and *ERBB3*, the first GWAS in this population has revealed *GATA3*, *BTN3A1*, and *SUOX* as additional genetic markers for T1D (17). However, the number of risk loci confirmed so far in Chinese patients with T1D is barely comparable to that in Caucasians, and several barriers have been in our way to pursue extra determinants of disease susceptibility. The first issue to be settled when handling genes with moderate effect sizes is a sufficiently large sample capacity to ensure statistical power. Second, as Chinese population is comprised of various ethnicities and certain ethnic minorities are genetically similar to Europeans instead of Han (18), findings generated from an admixed population can be confusing. Third, given that T1D may occur at any age and that the majority of new-onset T1D subjects in China are diagnosed during adulthood (19), we cannot obtain a full view of genetic architecture using data from studies restricted to childhood-onset T1D. Therefore, the considerably large collection of both T1D subjects and non-diabetic controls covering all age groups from genetically homogeneous Han population has enabled us to decipher the impact of *ACOXL* polymorphisms on T1D susceptibility in Chinese.

Though the genotype distribution of rs4849135 did not show a significant difference between the two groups (**Table 2**), the T allele did reduce the risk for T1D with an OR of 0.85, which was compatible with the result (OR 0.89) in GWASs conducted among Caucasians (4). In the dominant model, carrying the T allele was associated with lower odds of T1D compared with homozygotes of G allele. It was notable that in the recessive model, two copies of the T allele were not significantly associated with lower odds compared with individuals carrying 0–1 copy of the T allele, which could probably be explained by the mixture of the higher-risk G/G genotype with the lower-risk G/T genotype as well as the relatively low frequency of the T/T genotype observed in this population. These findings suggest that the ACOXL gene variant rs4849135 may be associated with T1D susceptibility, consistent with the findings reported in the ImmunoChip study. However, as no direct functional evidence exists, its role in T1D requires further investigation. Concerning rs4849165, after adjusting for age, the association with T1D was best explained by an additive model, suggesting a dose-dependent effect of the minor allele C. While the unadjusted analysis initially suggested a particularly strong effect for the homozygous C/C genotype, this was not statistically significant in the recessive model after accounting for age as a confounder.

The advent of overt T1D is usually preceded by the appearance of autoantibodies against islet (1). HLA complex and non-HLA loci both modify the specificity of the antibody setting in motion autoimmune process, namely, IAA or GADA as the first antibody to appear (20, 21). Before the diagnosis of classic T1D in our department, the patients were routinely tested for the three autoantibodies against islet. Nevertheless, neither of the two *ACOXL* gene polymorphisms showed a correlation with GADA, IA-2A, or ZnT8A no matter which genetic model was tested. Quite a number of genetic association studies in Caucasian patients with T1D are conducted in those with childhood onset, i.e., diagnosed between 1 and 14 years of age whereas the prevalence of adulthood-onset (≥18 years) T1D in Chinese should be worthy of note. We went on to separate the T1D participants into either group of early onset or those with late onset based on the two thresholds discussed above. It was found that patients carrying the T allele of rs4849135 were more frequently diagnosed at age ≥18 years than those with G/G genotype, which could at least explain in part why T1D with adulthood onset is relatively common in Chinese. On the other hand, rs4849165 did not appear to be a modulator of onset age for T1D in this population. We next sought to determine if the allelic variants of *ACOXL* had any impact on the beta-cell function of patients, for whom both FCP and PCP levels were measured. A previous study on the declining pattern of beta-cell function in Chinese patients with T1D defined both FCP <50 pmol/L and 2-h PCP <100 pmol/L as failure of beta-cell function (22). We paid particular attention to beta-cell failure which was unfavorable for the control of both hyperglycemia and complications and adopted the two cutoff values above to categorize patients of the current dataset. As a result, patients with the T1D-permissive C/C genotype of rs4849165 were more likely to experience failure of beta-cell function (PCP <100 pmol/L) compared with those carrying T/T or T/C genotype. In contrast, the T allele of rs4849135 was associated with a lower likelihood of beta-cell function failure compared with the G allele. Although we could not find data from other populations to substantiate the relationship between *ACOXL* and the C-peptide levels of T1D patients, increasing evidence was collected from multiple ethnicities to support the regulatory role of non-HLA SNPs such as vitamin D receptor gene, *PTPN22*, and *FCRL3* on the beta-cell function of subjects with T1D (23, 24). The main limitation of this research was that only cross-sectional profiles of patients such as autoantibody status and C-peptide concentrations could be presented here. The control group was not screened for islet autoantibodies; therefore, we cannot completely rule out the possibility that some individuals were in the preclinical stage of T1D. We were unable to figure out whether *ACOXL* polymorphisms alone or coupled with other risk loci could predict seroconversion to positivity of islet autoantibodies, progression to overt T1D, or the rapid decay of beta-cell function in Chinese. An additional limitation is that the exact timing of C-peptide measurement was not uniformly available; however, we included disease duration as a covariate in our analyses, which reduces the likelihood of bias due to the differential decline of C-peptide levels over time (25).

Aside from its association with T1D in Caucasian and in the Chinese population described here, genetic variants within *ACOXL* have also been involved in the pathogenesis of several diseases probably ascribed to either aberrant immunity or metabolic disorder. Alopecia areata was another autoimmune disease with evident genetic predisposition. Researchers from Columbia University integrated the data of two genome-wide association studies on alopecia areata and uncovered *ACOXL* rs3789129 reaching statistical significance, the association strength of which was second only to the HLA complex (14). Chronic lymphocytic leukemia was a malignancy of B lymphocyte encompassing a pronounced familial component. A meta-analysis exploited the genome-wide information of 2,343 European cases and 2,854 controls from 22 studies and identified nine new susceptibility loci including *ACOXL* rs13401811 for chronic lymphocytic leukemia (13). Since only a few of type 2 diabetes-related SNPs initially identified in populations of European ancestry could be replicated in African American which harbored a high genetic diversity, the GENNID Study Group managed to perform a linkage analysis utilizing the genotypes of 9,203 SNPs and established the association of 24 novel candidate loci such as *ACOXL* rs7583450 with type 2 diabetes in the African American subgroup (15). Additionally, syndromic obesity was defined by obesity with at least one other criterion like intellectual disability, facial dysmorphism, or congenital malformations. Vuillaume ML and her colleagues identified a list of new candidate loci in a cohort of 100 French children presented with syndromic obesity, and a vital branch of these genomic locations, e.g., *ACOXL*, was believed to engage in lipid metabolism (16). With a growing body of evidence gathered from epidemiological studies favoring the correlation between obesity and the risk for asthma, Zhu Z and his colleagues conducted a cross-trait GWAS enrolling 457,822 European participants from the UK Biobank in order to determine the shared genetic components between metabolic traits and subtypes of asthma. Consequently, *ACOXL* rs72836344 was found to be an overlapping variant between late-onset asthma and BMI (26). In this context, one might conceive that *ACOXL* could serve as a bridge between immunity and metabolism.

Research on *ACOXL* included but were not limited to genetic association studies and could be extended to the field of clinical relevance like treatment and prognosis for malignancies. Lipid metabolism was fundamental for the progression and metastasis of tumor. Jiang A and his colleagues developed a prognostic index for early-stage pulmonary adenocarcinoma according to the expression levels of six lipid metabolism-related genes, including *ANGPTL4*, *NPAS2*, *SLCO1B3*, *ACOXL*, *ALOX15*, and *B3GALNT1*. Patients with a higher prognostic index tended to have poorer overall survival and respond better to immune checkpoint inhibitors (27). Dong Y and his colleagues found that the transcriptomics of genes related to fatty acid metabolism such as *ACOXL* was valuable for classifying cutaneous melanoma into either hot or cold tumors, which showed distinct response rates for immunotherapy with checkpoint inhibitors (28). Additionally, Pontén F et al. carried out a proteomic analysis of human prostate and disclosed the potential of TMEM79 and ACOXL proteins to distinguish between benign and malignant prostatic tissues (29). In a recent study, Japanese scholars analyzed publicly available data of ChIP-Seq in various mouse tissues and discovered a T cell-specific enhancer-like region which was localized within the 9th intron of *ACOXL*, at around 200 kb upstream of *Bim* and at nearly 90 kb upstream of *Bub1.* This *cis*-regulatory enhancer was thus termed *E^BAB (Bub1-Acoxl-Bim)^*. In *E^BAB^* knockout mice, thymocytes harboring TCR with high affinity toward self-antigen were defective in apoptosis, which could be attributed to the insufficient expression of *Bim.* T cells which strongly recognized self-peptide and evaded negative selection in the thymus could subsequently result in autoimmunity (30). Their findings laid the groundwork for mechanistic studies on how changes in *ACOXL* could trigger autoimmune process in mice. These observations raise the possibility that *ACOXL* may influence immune tolerance through enhancer-mediated regulation of thymocyte apoptosis and negative selection, thereby indirectly affecting autoimmunity. In addition, given the reported associations of *ACOXL* variants with metabolic traits such as type 2 diabetes, obesity, and asthma, it is also possible that *ACOXL* polymorphisms contribute to T1D by modulating the pathways of lipid metabolism that secondarily impact beta-cell function. While these findings highlight the potential roles of *ACOXL* in immunity and metabolism, important limitations should be noted. *ACOXL* is primarily a peroxisomal fatty acid β-oxidation enzyme with limited expression in immune tissues and the pancreas, and there is no functional evidence supporting its causal role in autoimmunity. Therefore, the observed associations may reflect linkage disequilibrium with truly causal variants at the 2q13 locus rather than direct functional effects of *ACOXL*. Furthermore, the cross-sectional nature of this study precludes causal inference, and independent longitudinal studies are needed to validate our findings. Although all participants were Han Chinese, we cannot completely rule out regional genetic heterogeneity within this population. To mitigate this potential bias, recruitment was restricted to a single tertiary hospital in Hunan Province, which ensured a relatively homogeneous cohort. However, this strategy may also limit the generalizability of our findings to broader Chinese populations. Finally, environmental exposures known to influence T1D risk, such as viral infections and dietary factors, were not assessed in this study.

Our findings suggest that ACOXL variants may contribute not only to T1D susceptibility but also to clinical heterogeneity such as age at onset and beta-cell function in Chinese patients. These results raise the possibility that genes beyond classical immune pathways, including those involved in lipid metabolism, may modulate T1D pathogenesis and provide a basis for future mechanistic studies. Collectively, the data presented here have, for the first time, established the role of *ACOXL* gene polymorphisms in susceptibility to and the clinical features of T1D among the Chinese population. Whether these polymorphisms in combination with other genetic variants identified in this population can aid in the recognition of individuals genetically prone to T1D, the appearance of islet autoantibodies with distinct specificities and the development of full-blown T1D or rapid progressors to failure of beta-cell function should be a topic of interest for future studies with a longitudinal design.

## Data Availability

The original contributions presented in the study are included in the article/supplementary material. Further inquiries can be directed to the corresponding author.

## References

[1] Ilonen J Lempainen J Veijola R. The heterogeneous pathogenesis of type 1 diabetes mellitus. Nat Rev Endocrinol. (2019) 15:635–50. doi: 10.1038/s41574-019-0254-y, PMID: 31534209

[2] Cooper JD Smyth DJ Smiles AM Plagnol V Walker NM Allen JE. Meta-analysis of genome-wide association study data identifies additional type 1 diabetes risk loci. Nat Genet. (2008) 40:1399–401. doi: 10.1038/ng.249, PMID: 18978792 PMC2635556

[3] Ortega HI Udler MS Gloyn AL Sharp SA. Diabetes mellitus polygenic risk scores: heterogeneity and clinical translation. Nat Rev Endocrinol. (2025) 21:530–45. doi: 10.1038/s41574-025-01132-w, PMID: 40467969 PMC12614124

[4] Onengut-Gumuscu S Chen WM Burren O Cooper NJ Quinlan AR Mychaleckyj JC. Fine mapping of type 1 diabetes susceptibility loci and evidence for colocalization of causal variants with lymphoid gene enhancers. Nat Genet. (2015) 47:381–6. doi: 10.1038/ng.3245, PMID: 25751624 PMC4380767

[5] Sharp SA Rich SS Wood AR Jones SE Beaumont RN Harrison JW. Development and standardization of an improved type 1 diabetes genetic risk score for use in newborn screening and incident diagnosis. Diabetes Care. (2019) 42:200–7. doi: 10.2337/dc18-1785, PMID: 30655379 PMC6341291

[6] Erlich H Valdes AM Noble J Carlson JA Varney M Concannon P. HLA DR-DQ haplotypes and genotypes and type 1 diabetes risk: analysis of the type 1 diabetes genetics consortium families. Diabetes. (2008) 57:1084–92. doi: 10.2337/db07-1331, PMID: 18252895 PMC4103420

[7] Luo S Lin J Xie Z Xiang Y Zheng P Huang G. HLA genetic discrepancy between latent autoimmune diabetes in adults and type 1 diabetes: LADA China study no. 6. J Clin Endocrinol Metab. (2016) 101:1693–700. doi: 10.1210/jc.2015-3771, PMID: 26866570

[8] Pei Z Chen X Sun C Du H Wei H Song W. A novel single nucleotide polymorphism in the protein tyrosine phosphatase N22 gene (PTPN22) is associated with Type 1 diabetes in a Chinese population. Diabetes Med. (2014) 31:219–26. doi: 10.1111/dme.12331, PMID: 24117662

[9] Jin P Xiang B Huang G Zhou Z. The association of cytotoxic T-lymphocyte antigen-4 + 49A/G and CT60 polymorphisms with type 1 diabetes and latent autoimmune diabetes in Chinese adults. J endocrinological Invest. (2015) 38:149–54. doi: 10.1007/s40618-014-0162-x, PMID: 25185645

[10] Bi C Li B Cheng Z Hu Y Fang Z Zhai A. Association study of STAT4 polymorphisms and type 1 diabetes in Northeastern Chinese Han population. Tissue Antigens. (2013) 81:137–40. doi: 10.1111/tan.12057, PMID: 23360093

[11] Sun C Wei H Chen X Zhao Z Du H Song W. ERBB3-rs2292239 as primary type 1 diabetes association locus among non-HLA genes in Chinese. Meta gene. (2016) 9:120–3. doi: 10.1016/j.mgene.2016.05.003, PMID: 27331016 PMC4908278

[12] Van Veldhoven PP. Biochemistry and genetics of inherited disorders of peroxisomal fatty acid metabolism. J Lipid Res. (2010) 51:2863–95. doi: 10.1194/jlr.R005959, PMID: 20558530 PMC2936746

[13] Berndt SI Skibola CF Joseph V Camp NJ Nieters A Wang Z. Genome-wide association study identifies multiple risk loci for chronic lymphocytic leukemia. Nat Genet. (2013) 45:868–76. doi: 10.1038/ng.2652, PMID: 23770605 PMC3729927

[14] Betz RC Petukhova L Ripke S Huang H Menelaou A Redler S. Genome-wide meta-analysis in alopecia areata resolves HLA associations and reveals two new susceptibility loci. Nat Commun. (2015) 6:5966. doi: 10.1038/ncomms6966, PMID: 25608926 PMC4451186

[15] Hasstedt SJ Highland HM Elbein SC Hanis CL Das SK. Five linkage regions each harbor multiple type 2 diabetes genes in the African American subset of the GENNID Study. J Hum Genet. (2013) 58:378–83. doi: 10.1038/jhg.2013.21, PMID: 23552671 PMC3692593

[16] Vuillaume ML Naudion S Banneau G Diene G Cartault A Cailley D. New candidate loci identified by array-CGH in a cohort of 100 children presenting with syndromic obesity. Am J Med Genet Part A. (2014) 164a:1965–75. doi: 10.1002/ajmg.a.36587, PMID: 24782328

[17] Zhu M Xu K Chen Y Gu Y Zhang M Luo F. Identification of novel T1D risk loci and their association with age and islet function at diagnosis in autoantibody-positive T1D individuals: based on a two-stage genome-wide association study. Diabetes Care. (2019) 42:1414–21. doi: 10.2337/dc18-2023, PMID: 31152121

[18] Liu S Huang S Chen F Zhao L Yuan Y Francis SS. Genomic analyses from non-invasive prenatal testing reveal genetic associations, patterns of viral infections, and Chinese population history. Cell. (2018) 175:347–59.e14. doi: 10.1016/j.cell.2018.08.016, PMID: 30290141

[19] Weng J Zhou Z Guo L Zhu D Ji L Luo X. Incidence of type 1 diabetes in China, 2010-13: population based study. BMJ. (2018) 360:j5295. doi: 10.1136/bmj.j5295, PMID: 29298776 PMC5750780

[20] Lempainen J Laine AP Hammais A Toppari J Simell O Veijola R. Non-HLA gene effects on the disease process of type 1 diabetes: From HLA susceptibility to overt disease. J Autoimmun. (2015) 61:45–53. doi: 10.1016/j.jaut.2015.05.005, PMID: 26074154

[21] Ilonen J Hammais A Laine AP Lempainen J Vaarala O Veijola R. Patterns of β-cell autoantibody appearance and genetic associations during the first years of life. Diabetes. (2013) 62:3636–40. doi: 10.2337/db13-0300, PMID: 23835325 PMC3781470

[22] Li X Cheng J Huang G Luo S Zhou Z. Tapering decay of β-cell function in Chinese patients with autoimmune type 1 diabetes: A four-year prospective study. J Diabetes. (2019) 11:802–8. doi: 10.1111/1753-0407.12907, PMID: 30767397

[23] Habibian N Amoli MM Abbasi F Rabbani A Alipour A Sayarifard F. Role of vitamin D and vitamin D receptor gene polymorphisms on residual beta cell function in children with type 1 diabetes mellitus. Pharmacol reports: PR. (2019) 71:282–8. doi: 10.1016/j.pharep.2018.12.012, PMID: 30826568

[24] Pawłowicz M Filipów R Krzykowski G Stanisławska-SaChadyn A Morzuch L Kulczycka J. Coincidence of PTPN22 c.1858CC and FCRL3 -169CC genotypes as a biomarker of preserved residual β-cell function in children with type 1 diabetes. Pediatr Diabetes. (2017) 18:696–705. doi: 10.1111/pedi.12429, PMID: 27615679

[25] Ludvigsson J Carlsson A Deli A Forsander G Ivarsson SA Kockum I. Decline of C-peptide during the first year after diagnosis of Type 1 diabetes in children and adolescents. Diabetes Res Clin practice. (2013) 100:203–9. doi: 10.1016/j.diabres.2013.03.003, PMID: 23529064

[26] Zhu Z Guo Y Shi H Liu CL Panganiban RA Chung W. Shared genetic and experimental links between obesity-related traits and asthma subtypes in UK Biobank. J Allergy Clin Immunol. (2020) 145:537–49. doi: 10.1016/j.jaci.2019.09.035, PMID: 31669095 PMC7010560

[27] Jiang A Chen X Zheng H Liu N Ding Q Li Y. Lipid metabolism-related gene prognostic index (LMRGPI) reveals distinct prognosis and treatment patterns for patients with early-stage pulmonary adenocarcinoma. Int J Med Sci. (2022) 19:711–28. doi: 10.7150/ijms.71267, PMID: 35582412 PMC9108406

[28] Dong Y Zhao Z Simayi M Chen C Xu Z Lv D. Transcriptome profiles of fatty acid metabolism-related genes and immune infiltrates identify hot tumors for immunotherapy in cutaneous melanoma. Front Genet. (2022) 13:860067. doi: 10.3389/fgene.2022.860067, PMID: 36199579 PMC9527329

[29] O’Hurley G Busch C Fagerberg L Hallström BM Stadler C Tolf A. Analysis of the human prostate-specific proteome defined by transcriptomics and antibody-based profiling identifies TMEM79 and ACOXL as two putative, diagnostic markers in prostate cancer. PLoS One. (2015) 10:e0133449. doi: 10.1371/journal.pone.0133449, PMID: 26237329 PMC4523174

[30] Hojo MA Masuda K Hojo H Nagahata Y Yasuda K Ohara D. Identification of a genomic enhancer that enforces proper apoptosis induction in thymic negative selection. Nat Commun. (2019) 10:2603. doi: 10.1038/s41467-019-10525-1, PMID: 31197149 PMC6565714

